# Therapeutic role of mesenchymal stem cell-derived exosomes in respiratory disease

**DOI:** 10.1186/s13287-022-02866-4

**Published:** 2022-05-12

**Authors:** Mehdi Jahedi Zargar, Saeid Kaviani, Mohammad Vasei, Mina Soufi Zomorrod, Saeed Heidari Keshel, Masoud Soleimani

**Affiliations:** 1 Department of Hematology, Faculty of Medical Sciences, Tarbiat Modares University, Tehran, Iran; 2 Cell Therapy Based Research Center, Digestive Disease Research Institute, Shariati Hospital, Tehran University of Medical Sciences, Tehran, Iran; 3 Applied Cell Science and Hematology Department, Faculty of Medical Science, Tarbiat Modares University, Tehran, Iran; 4 Department of Tissue Engineering and Applied Cell Science, School of Advanced Technologies in Medicine, Shahid Beheshti University of Medical Sciences, Tehran, Iran

**Keywords:** Cell therapy, Exosome, MSC-derived exosomes, Lung diseases

## Abstract

Exosomes are extracellular vesicles found in various tissues, blood circulation, and tissue fluids, secreted into the extracellular environment by fusing a multivesicular body with a plasma membrane. Various cell types release these vesicles to contribute to many cellular functions, including intercellular communication, cell proliferation, differentiation, angiogenesis, response to stress, and immune system signaling. These natural nanoparticles have therapeutic effects in various diseases and exhibit a behavior similar to the cell from which they originated. In the meantime, exosomes derived from mesenchymal stem cells have attracted the attention of many researchers and physicians due to their unique ability to modulate the immune system, repair tissue and reduce inflammation. Numerous clinical and preclinical studies have examined the effect of MSC-derived exosomes in various diseases, and their results have been published in prestigious journals. This review article discusses the biogenesis and sources of exosomes, MSC-derived exosomes, the use of these exosomes in regenerative medicine, and treatments based on exosomes derived from stem cells in respiratory diseases.

## Introduction

Mesenchymal stem cells (MSCs) have unique biological properties due to their stem cell nature. These cells can regenerate themselves and differentiate into multiple cells [1]. Mesenchymal stem cells are isolated from various tissues and are widely distributed throughout the body, including bone marrow and adipose tissue [2]. Identification features of human mesenchymal stem cells, including adhesion capability in traditional culture, expression of CD105, CD73, and CD90, non-expression of CD45, CD34, CD14, CD11b, CD79a, CD19, and HLA-DR, differentiation into osteoblasts, adipocytes, and chondrocytes in vitro, have been expressed by International Association of Cell Therapy [3]. The significant capability of MSCs to proliferate in vitro and differentiate into different cells introduces these cells as therapeutic agents for regenerating necrotic cells or for connective tissue apoptosis. Mesenchymal stem cells can be differentiated into several classes, including adipocytes, endothelial cells, cardiomyocytes, chondrocytes, osteoblasts, and various cells like hepatocytes and neuron-like cells [4, 5]. Mesenchymal stem cells have low immunogenicity because of the lowly expression of MHC-I and the expression of a small number of MHC-II molecules [6, 7]. MSCs have also shown immune system modulation and regeneration capacity in various disease models [8–11]. At present, significant advances have been made in stem cell technology with good therapeutic prospects for treating different diseases such as respiratory diseases [12]. Many studies indicate that MSCs can affect activation, proliferation, and differentiation of cells acting on natural killer cells (NK), dendritic cells (DCS), macrophages, B lymphocytes, and T lymphocytes [13–16]. MSC migrate to the inflammation site through adhesion molecules and integrins such as VCAM-1 and VLA-4, affecting the damaged tissue through cell–cell contact and secretion of various trophic factors [17]. In a clinical trial on patients with cirrhosis, it was shown that mesenchymal stem cells were trapped in the lungs in the early hours after injection into peripheral blood and that they left the lungs after 48 h, migrated to the liver and spleen, and remained in these tissues for several days [18]. Other investigations have indicated that most injected MSCs are commonly trapped in the liver, spleen, and lungs and that a small number of these cells reach the damaged site. Therefore, focusing on cell-free therapies is of high importance [19]. MSCs secrete soluble factors such as growth factors, cytokines, and chemokines, and they also release extracellular vesicles (EVs), leading to therapeutic consequences through the exchange of cytoplasm and genetic material [20, 21]. The therapeutic ability of MSCs may depend on paracrine factors in the vesicles [22]. This paper will examine exosomes (especially MSC-derived ones) and their application in diagnosing and treating lung diseases.

### Biogenesis and sources of exosomes

Extracellular vesicles (EVs) are released from various cellular sources and have been known as messengers of cellular transmission through the delivery of lipids, proteins, and biologically active RNAs. EVs are separated into three subtypes: exosomes, microvesicles, and apoptotic bodies [23]. Among these extracellular vesicles, exosomes play an essential role in modulating the immune system, as well as in cellular communication [24]. Exosomes from MSCs show similar natural activity to these cells by encompassing and transporting functional biomolecules such as peptides, proteins, and RNA species to damaged tissues and cells [25]. In terms of mechanisms and cellular composition, exosomes resemble the cells derived from them [26]. Exosomes can be classified according to their cell or tissue of origin based on cellular elements and protein content. Proteins of cell-derived exosomes typically include integrins, adhesive molecules, MHC I and II, transferrin receptors, and other cell surface exosomes. Nonspecific proteins of exosomes restricted to plasma membranes, cytosols, and endosomal elements, including fusion and transporter proteins, and, cytoskeletal proteins, heat shock proteins, contribute to multivesicular and other cellular processes [27]. Studies have indicated that exosomes derived from endosomes recreate a necessary function in cell-to-cell communication. Exosomes are discovered in most biological fluids, including serum, breast milk, saliva, urine, synovial fluid, amniotic fluid, lymph, bile, gastric acid, tears, and cerebrospinal fluid (CSF) produced in a variety of cells. In addition to cellular communication, exosomes are involved in tumor progression, myelin formation, and cell maintenance [28].

### Exosomes originated from mesenchymal stem cells

**Fig. 1:**
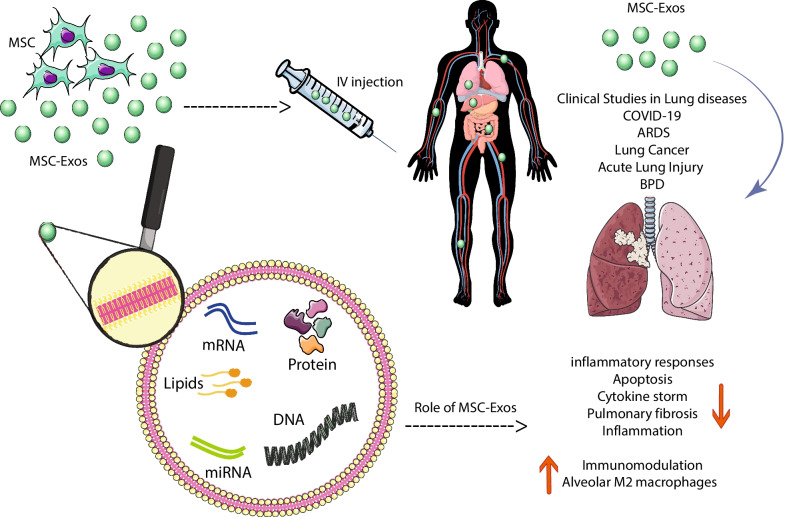
The schematic role of MSC exosomes in respiratory diseases

Mesenchymal stem cells from different sources have received significant attention as an alternative treatment for various rare diseases due to their ability to stimulate tissue regeneration and modulate active immune cells [29, 30]. These cells act in various diseases through cell–cell contact and environmental changes induced by the release of soluble factors [31]. MSC-derived exosomes have a crucial role in the function of mesenchymal stem cells as stromal support cells responding to external stimuli and maintaining tissue homeostasis. At injury or disease, tissue homeostasis is impaired, and exosomes’ key role becomes apparent. MSC-derived exosomes are affluent in biologically active molecules like proteins and RNAs and can adequately play their role [32]. Exosomes contain large amounts of membrane and cytoplasmic proteins such as extracellular matrix proteins, receptors, enzymes, transcription factors, nucleic acids, and lipids [33] (Fig. 1). MSC exosomes express CD markers such as CD73, CD44, CD29, and CD105 [34] and include proteins, mRNAs, and microRNAs transported to receptor cells and change the manners of neighboring cells [35]. MSC-derived exosomes represent adhesion molecules (FN1, EZR, IQGAP1, CD47, integrin, and LGALS1/LGALS3), receptors (PDGFRB, EGFR, and PLAUR), signaling molecules (RRAS/NRAS, MAPK1, GNA13/GNG12, CDC42, and VAV2), and antigens related to MSCs (CD63, CD63, CD81, CD109, CD151, CD248, and CD276) [36]. MSC exosomes contribute to cellular functions, including proliferation, adhesion, transcription, migration, and differentiation [35]. MSC-derived exosomes inhibit inflammation, induce angiogenesis, prevent fibrosis, increase neuronal survival and differentiation, stimulate ECM regeneration and modulate immune cells [37].

Studies have shown that MSC-derived exosomes comprise more than 850 and 150 gene products and miRNAs, respectively [38, 39], which are applied in biological processes such as organism growth, immune modulation (miR-155 and miR-146), epigenetic regulation, tumorigenesis, and tumor advance (miR-23b, 451miR-, miR-223, miR-24, miR-125b, miR-31, miR-214, and miR-122) [40, 41]. These exosomes contain growth factors and cytokines including IL-10, TGFβ1, IL-6, and hepatic growth factor (HGF), which are involved in immune system modulation [42]. In addition, studies have demonstrated that MSC-derived exosomes have a pivotal role in promoting angiogenesis and tissue repair through factors such as vascular endothelial growth factor (VEGF), extracellular matrix metalloproteinase inhibitor (EMMPRIN), and matrix metallopeptidase 9 (MMP-9) [43].

### Utilization of MSC-derived exosomes in regenerative medicine

MSCs have the fantastic possibility to sustain tissue homeostasis because these cells can enter into damaged tissue and regulate the immune system and tissue regeneration through cellular and molecular processes [44–46]. Further studies have revealed that the beneficial effects of MSCs in repair are related to paracrine signaling, including secreted vesicles such as exosomes [47–50]. Various studies show that exosomes secreted by MSCs can replace stem cell-based therapies in different models of injury and disease [51, 52]. The therapeutic effects of MSC exosomes have been demonstrated in preclinical studies in various diseases such as CVD, renal, hepatic, and neurological diseases, healing of wounds, and other diseases [53]. We briefly review some of these investigations.

A study by Cui et al. on exosomes derived from adipose tissue MSCs in disease showed that these exosomes caused a significant increase in survival of H9C2 cell line under hypoxia/re-oxygenation (H/R) conditions in vitro. In this study, administration of Ad-MSC-derived exosomes via the Wnt/β-catenin signaling pathway is protected against myocardial ischemia in vivo [54]. In another research on a model of myocardial ischemic injury, exosomes from BM-derived mesenchymal stem cells reduced apoptosis and the size of myocardial infarction and, after that, recovered heart function by persuading cardiac autophagy via two pathways such as AMPK/mTOR and Akt/mTOR routes [55]. MSCs have revealed promising results in acute and chronic kidney damage. A study on a rat model of renal damage revealed that intravascular injection of human umbilical cord-derived mesenchymal stem cells (huMSCs) in a mouse model with ischemia–reperfusion injury (IRI) of the kidney increases renal vein density, reducing renal fibrosis by direct transfer of vascular endothelial growth factor, a process in which mRNAs are involved [56]. Another study showed that an intrarenal injection of adipose tissue-derived EVs in a swine model with renal artery stenosis reduced the level of proinflammatory factors, including TNF-α, IL-6, and IL-1β, increased IL-10 levels in renal vein and decreased kidney inflammation, indicating the immune-modulating capacity of EVs by changing proinflammatory to tubular repair macrophages [57]. Effects such as protecting the liver tissue of exosomes separated from human embryonic stem cell-derived mesenchymal stem cells (hESC-MSCs) were investigated in a model of acute liver injury [58].

Moreover, it was found that these exosomes contribute to the regeneration of damaged liver tissue through positive regulation of PCNA expression, cyclin D1 cell cycle regulator, and Bcl-xL anti-apoptotic gene [58]. In another research, Li et al. assessed exosomes derived from human umbilical cord mesenchymal stem cells (hUMSC) and showed that these exosomes improve liver fibrosis by inhibiting EMT of liver cells and producing collagen, as well as recovering aspartate aminotransferase activity in serum and inactivating TGF-β1/Smad2 pathway [59]. One of the most prominent outcomes of MSC-derived exosomes is their ability to transit the blood–brain barrier (BBB) and reach the brain parenchyma. Therapeutic advantages of MSC-derived exosomes in the cure of neurodegenerative disorders have been demonstrated in different investigations. Exosomes targeted against α-synuclein reduce mRNA and alpha-synuclein protein levels in the brain [60, 61]. Studies on BM-MSC exosomes have indicated that these exosomes contain miR-133b, which leads to neurite regeneration and improves stroke in mouse models [62]. Intravenous injection of BM-MSC exosomes in a mouse stroke model increased neurovascular flexibility and improved axon density in the ischemic margin region of the brain [63]. Exosomes derived from Wharton Jelly mesenchymal stem cells lead to angiogenesis in vivo and enhance wound healing via the Wnt4 pathway and activation of β-catenin [64]. MiRNAs such as miR-21, miR-23a, miR-125b, and miR-145 originating from WJ-MSC exosomes contribute to wound healing by inhibiting scar formation and myofibroblast accumulation and reducing collagen deposition [65]. Another study found that exosomes derived from BM-MSC via Akt, ERK, and STAT3 signaling pathways could raise fibroblast proliferation, migration and increase HGF, IGF1, NGF, and SDF1 levels [66]. Exosomes derived from Wharton Jelly-MSC showed significant medicinal effects by improving bronchopulmonary dysplasia, pneumonia, pulmonary hypertension, fibrosis, and regulating the phenotype of pulmonary macrophages in the lung tissue [67].

Studies have shown that microRNAs from exosomes derived from mesenchymal stem cells, such as miR-125a-3p, improve Treg survival and prevent T cells from differentiating into effector cells [68]. These microRNAs, including miR-146a, function in inflammatory responses using the NF-κB signaling pathway [69]. They have debilitating effects on dendritic cells through miR-21-5p, miR-142-3p, miR-223-3p, and miR 126-3p [70]. In addition, they inhibit the production of cytokines such as IL-6 through miR-142-3p [70].

### MSC-derived exosomes-based therapies in respiratory disease

Clinical trials based on anti-inflammatory medications combined with glucocorticoids have failed to treat lung diseases [71, 72]. Preclinical studies indicate that MSC-derived exosomes have a significant healing prospect in the regeneration and rehabilitation of several lung diseases via various molecular pathways and by influencing lung tissue target cells like immune cells, endothelial and epithelial cells [73, 74]. MSC-derived exosomes are promising for the cure of diverse lung diseases, including idiopathic pulmonary fibrosis (IPF), ALI, ARDS, pulmonary artery hypertension, asthma, pneumonia, inflammatory lung disease, silicosis, chronic obstructive pulmonary disease (COPD), and Bronchopulmonary dysplasia [75]. While the production of exosomes is practically and economically tricky compared to that of MSCs, they are not trapped in the lungs as MSCs injected intravenously do, have advantages such as small size (approximately 100 nm), and can be favorable as aerosol inhalation for the treatment of airborne diseases [76] (Fig. 1). Proinflammatory mechanisms are inhibited by MSC-derived exosomes and are associated with remodeling of inflammatory lung disease and reduction in oxidative stress and pulmonary fibrosis [77].

One study used exosomes derived from human BM mesenchymal stem cells in a bleomycin-induced pulmonary fibrosis model. This study demonstrated the use of exosomes as an exciting and innovative approach to treating fibrotic lung disease. Exosomes improve pulmonary fibrosis by modulating the monocyte phenotype [78]. In a study by Ahn et al., VEGF was shown to be highly important in protecting exosomes in pulmonary hyperoxia damages. Exosomes derived from umbilical cord mesenchymal stem cells have significant efficacy in improving impaired alveolar function through angiogenic effects, reducing apoptosis, and limiting macrophages and inflammatory responses in a mouse model of lung injury [74]. In another study, mitochondrial MSC exosomes increased the production of alveolar M2 macrophages to reduce acute lung damage and inhibit inflammatory cytokines [79]. Intravenous injection of MSC exosomes exhibits immunomodulatory impacts in bacterial pneumonia lesions by increasing monocytes’ phagocytic capacity and decreasing the secretion of inflammatory cytokines. Exosomes restore the metabolism of alveolar type 2 epithelial cells by raising intracellular ATP levels [80]. MSC-MVs stopped endothelial cell apoptosis via rising IL-10 levels and decreasing IL-6 expression in endothelial cell culture medium through HGF factor in vitro [81]. In an animal model of chronic obstructive pulmonary disease (COPD) study, a comparison was made between the healing capacity of hUC-MSCEVs and hUC-MSCs in remedy.

Both MSCs and exosomes derived from them improved peribronchial and vascular inflammation, thereby reducing the thickening of the alveolar septum in COPD via reducing the production of zeta C kinase protein and NF-κB subunits of p50 and p65 subunits [82]. MSC-derived exosomes are effective, promising treatments for ALI/ARDS. In one study, human BM was injected by chips using MSCs into an ALI model. This research observed a significant decrease in macrophage-2 inflammatory protein levels in Bronchoalveolar lavage fluid (BALF), pneumonia, neutrophil infiltration, and protein penetrance [83]. In addition, a study by Abreu et al. [84] found that exosomes derived from HBM-MSCs could help reduce inflammatory responses in ARDS through mitochondrial transmission. Studies have shown that the injection of BM-MSC-derived exosomes can prevent myofibroblastic differentiation associated with TGF-β1 in pulmonary fibrosis [85]. Despite the COVID-19 pandemic, MSC-derived exosomes can be good as a therapeutic agent in this disease and its associated difficulties, such as acute lung injury and ARDS.

As a therapeutic strategy for severe COVID-19, exosomes are used with convalescent plasma since it contains acquired immune antibodies that require consideration that it contains trillions of exosomes. These exosomes are produced by immunomodulatory cells that transmit miRNAs [86]. MSC-derived exosomes have remarkable characteristics, including antiviral properties, immune system regulation, and tissue repair. A present investigation showed that MSC-derived exosomes could replace MSCs because they are similar to mesenchymal stem cells in COVID-19 [87]. In a study of severe COVID-19 disease, exosomes derived from allogeneic BM-MSCs were used in 24 patients, which showed that MSC exosomes could be a potent therapeutic candidate for treating severe COVID-19 [88]. Because the pathogenesis of SARS-CoV-2 is equivalent to many viruses and results in complications such as ARDS and lung damage, treatment approaches launched on MSCs or MSC-derived exosomes were examined in SARS-CoV-2. In previous studies, MSC-derived exosomes have been shown a favorable reaction to ARDS and suppress cytokine storms by transmitting mRNA and miRNA to lung tissues [23, 89]. The schematic role of MSC exosomes in respiratory diseases is depicted in Fig. 1.

In recent years, clinical studies on the use of exosomes in lung diseases such as COVID-19, ARDS, Early-staged Lung Cancer, Acute Lung Injury and BPD have been documented, as shown in Table 1.

**Table 1:** Clinical trials in Exosomes Therapy In Respiratory Diseases

No	Title and sponsor	Trial ID	Location	Design	Primary outcome	Recruitment status	Phase
1.	The use of exosomes for the treatment of acute respiratory distress syndrome or novel coronavirus pneumonia caused by COVID-19 (ARDOXSO) Sponsor: AVEM HealthCare	NCT04798716	United States, California	Open label, interventional, mesenchymal stem cell exosomes for the treatment of COVID-19 positive patients with acute respiratory distress syndrome and/or novel coronavirus pneumonia N:55	Measure and report the number of participants with treatment-related-adverse events Tabulate and report the number of IMV days for patients receiving ARDOXSO™ perinatal MSC-derived exosome therapy	Not yet recruiting July 21, 2021	Phase 1 Phase 2
2.	Safety and efficiency of method of exosome inhalation in COVID-19 associated pneumonia (COVID-19EXO2) Sponsor: Olga Tyumina	NCT04602442	Russian, Samara	Randomized, interventional, the extended protocol of evaluation of safety and efficiency of method of exosome inhalation in COVID-19 associated two-sided pneumonia N: 90	Number of participants with non-serious and serious adverse events during trial Number of participants with non-serious and serious adverse during inhalation procedure	Enrolling by invitation October 26, 2020	Phase 2
3.	Evaluation of safety and efficiency of method of exosome inhalation in SARS-CoV-2 associated pneumonia. (COVID-19EXO) Sponsor: State-Financed Health Facility “Samara Regional Medical Center Dinasty”	NCT04491240	Russian, Samara	Randomized, interventional, the protocol of evaluation of safety and efficiency of method of exosome inhalation in SARS-CoV-2 associated two-sided pneumonia N: 30	Number of participants with non-serious and serious adverse events during trial Number of participants with non-serious and serious adverse during inhalation procedure	Completed November 4, 2020	Phase 1 Phase 2
4.	A safety study of IV stem cell-derived extracellular vesicles (UNEX-42) in preterm neonates at high risk for BPD Sponsor: United Therapeutics	NCT03857841	United States, Colorado, Massachusetts, Mississippi, Missouri	Randomized, interventional, a safety study of intravenous infusion of bone marrow mesenchymal stem cell-derived extracellular vesicles (UNEX-42) in preterm neonates at high risk for bronchopulmonary dysplasia N: 3	Number of subjects with treatment-emergent adverse events during the post-treatment phase	Terminated October 12, 2021	Phase 1
5.	A clinical study of mesenchymal stem cell exosomes nebulizer for the treatment of ARDS Sponsor: Ruijin Hospital	NCT04602104	China, Shanghai	Randomized, double-blinded, controlled clinical study of allogeneic human mesenchymal stem cell exosomes (hMSC-Exos) nebulized inhalation in the treatment of acute respiratory distress syndrome N:169	Incidence of adverse reaction Time to clinical improvement 28-day mortality	Recruiting November 2, 2021	Phase 1 Phase 2
6.	A tolerance clinical study on aerosol inhalation of mesenchymal stem cells exosomes in healthy volunteers Sponsor: Ruijin Hospital	NCT04313647	China, Shanghai	Open label, non randomized, interventional, a tolerance clinical study on aerosol inhalation of mesenchymal stem cells exosomes in healthy volunteers N: 24	Number of Participants With Adverse Reaction (AE) and severe adverse reaction (SAE)	Completed August 4, 2021	Phase 1
7.	Omics sequencing of exosomes in body fluids of patients with acute lung injury Sponsor: Nanfang Hospital of Southern Medical University	NCT05058768	China, Guangdong	Observational, case–control, exosomes in urine, blood, and alveolar lavage fluid from patients with acute respiratory distress syndrome (ADRS) were sequenced by omics N:180	Compare the omics differences of blood samples between the experimental and control groups Compare the omics differences of urine samples between the experimental and control groups	Recruiting September 28, 2021	Case–Control
8.	Exosomes derived from placental mesenchymal stem cells as treatment for severe COVID-19: Phase 1 and 2 clinical trials Sponsor: Omid Cell and Tissue center	IRCT20200413047063N2	Tehran, Iran	Participants were randomly divided into two equal groups using a randomized double AB blocking method based on a random number table. Patients allocated randomly to two groups: (1) Intervention 1, Patients will receive Six doses of Exosomes. (2) Control, Patients will receive conventional therapy N:50	Adverse events assessment	Recruiting July 8, 2021	Phase 1 Phase 2
9.	Molecular profiling of exosomes in tumor-draining vein of early-staged lung cancer (ExOnSite-Pro) Sponsor: University Hospital, Limoges	NCT04939324	France, Limoges	Open label, single group assignment, Analyse du Profil moléculaire Des Exosomes de la Veine Pulmonaire Dans le Cancer Bronchique de Stade précoce N:30	Evaluate size distribution, concentration and molecular profiling of pulmonary vein exosomes at inclusion	Recruiting November 11, 2021	Not applicable
10.	A pilot clinical study on inhalation of mesenchymal stem cells exosomes treating severe novel coronavirus pneumonia Sponsor: Ruijin Hospital	NCT04276987	China, Shanghai	Open label, single group assignment, a pilot clinical study on aerosol inhalation of the exosomes derived from allogenic adipose mesenchymal stem cells in the treatment of severe patients with novel coronavirus pneumonia N:24	Adverse reaction (AE) and severe adverse reaction (SAE) Time to clinical improvement (TTIC)	Completed September 7, 2020	Phase 1
11.	Extracellular vesicle infusion treatment for COVID-19 Associated ARDS (EXIT-COVID19) Sponsor: Direct Biologics, LLC	NCT04493242	United States, Alabama, California, Pennsylvania, Texas	Randomized, double-blinded, bone marrow mesenchymal stem cell derived extracellular vesicles infusion treatment for COVID-19 associated acute respiratory distress syndrome (ARDS): a phase II clinical trial N: 120	7 day change in partial pressure of arterial oxygen to fraction of inspired oxygen ratio	Completed December 6, 2021	Phase 2
12.	Safety and efficacy of exosomes overexpressing CD24 in two doses for patients with moderate or severe COVID-19 Sponsor: Athens Medical Society	NCT04902183	Greece, Athens, Attica	Randomized, single, a Phase II randomized, single-blind dose study to evaluate the safety and efficacy of exosomes overexpressing CD24 in 10^9 dose versus 10^10 dose, for the prevention of clinical deterioration in patients with moderate or severe COVID-19 N:90	Collection of serious adverse events Proportion of patients related with Respiratory rate and SpO2 saturation	Recruiting June 15, 2021	Phase 2

## Conclusion

Clinical and preclinical studies have shown advantageous effects of MSC-derived exosomes. Since stem cell therapy is associated with clinical challenges such as high cell count, selective dose, cell injection routes, cell safety, exosomes derived from these cells have become highly important in various diseases. Exosomes have received much attention in biomarker research today and are even regarded as an alternative strategy for stem cell-based regenerative therapies. For this purpose, the use of separation methods and optimization of these exosomes can be promising in clinical studies of various diseases, including lung diseases.

## Data Availability

Not applicable.

## Abbreviations

PF: Pulmonary fibrosis
IPF: Idiopathic pulmonary fibrosis
BALF: Bronchoalveolar lavage fluid
COPD: Chronic obstructive pulmonary disease
ATII: Alveolar cell type 2
MSCs: Mesenchymal stem cells
PD-MSCs: Placenta-derived mesenchymal stem cells
HBM-MSC: Human bone marrow-derived mesenchymal stem cell
hUMSC: Human umbilical cord mesenchymal stem cells
huMSCs: Human umbilical cord-derived mesenchymal stem cells
WJ-MSC: Wharton’s jelly-derived mesenchymal stem cells
AD-MSC: Adipose tissue-derived mesenchymal stem cell
hESC-MSCs: Human embryonic stem cell-derived mesenchymal stem cells
EVs: Extracellular vesicles
HLA: Human leukocyte antigen
BBB: Blood–brain barrier
CSF: Cerebrospinal fluid
VEGF: Vascular endothelial growth factor
TGF-b1: Transforming growth beta-1
IL-10: Interleukin 10
IL-6: Interleukin 6
IL-1β: Interleukin 1β
IGF-1: Insulin-like growth factor 1
NGF: Nerve growth factor
EMMPRIN: Extracellular matrix metalloproteinase inhibitor
HGF: Hepatocyte growth factor
NK cell: Natural killer cells
DCS: Dendritic cells
PD-L1: Programmed death ligand 1
MMPs: Matrix metalloproteases
H/R: Hypoxia/Re-oxygenation
CVD: Cardiovascular disease
